# Integrated high-throughput analysis identifies super enhancers associated with chemoresistance in SCLC

**DOI:** 10.1186/s12920-019-0520-9

**Published:** 2019-05-22

**Authors:** Jiarong Bao, Man Li, Shumei Liang, Yunchu Yang, Jingfang Wu, Qingqing Zou, Shun Fang, Size Chen, Linlang Guo

**Affiliations:** 1 Department of Pathology, Zhujiang Hospital, Southern Medical University, 253 Gongye Road, Guangzhou, 510282 People’s Republic of China; 2 Department of Pathology, The Fifth Affiliated Hospital, Southern Medical University, Guangzhou, 510900 People’s Republic of China; 3 Department of Oncology, The First Affiliated Hospital of Guangdong Pharmaceutical University, Guangzhou, 510080 People’s Republic of China

**Keywords:** Small cell lung cancer, Chemoresistance, Super enhancer

## Abstract

**Background:**

Chemoresistance is a primary clinical challenge for the management of small cell lung cancer. Additionally, transcriptional regulation by super enhancer (SE) has an important role in tumor evolution. The functions of SEs, a key class of noncoding DNA *cis*-regulatory elements, have been the subject of many recent studies in the field of cancer research.

**Methods:**

In this study, using chromatin immunoprecipitation-sequencing and RNA-sequencing (RNA-seq), we aimed to identify SEs associated with chemoresistance from H69AR cells. Through integrated bioinformatics analysis of the MEME chip, we predicted the master transcriptional factors (TFs) binding to SE sites and verified the relationships between TFs of SEs and drug resistance by RNA interference, cell counting kit 8 assays, quantitative real-time reverse transcription polymerase chain reaction.

**Results:**

In total, 108 SEs were screened from H69AR cells. When combining this analysis with RNA-seq data, 45 SEs were suggested to be closely related to drug resistance. Then, 12 master TFs were predicted to localize to regions of those SEs. Subsequently, we selected forkhead box P1 (FOXP1), interferon regulatory factor 1 (IRF1), and specificity protein 1 (SP1) to authenticate the functional relationships of master TFs with chemoresistance via SEs.

**Conclusions:**

We screened out SEs involved with drug resistance and evaluated the functions of FOXP1, IRF1, and SP1 in chemoresistance. Our findings established a large group of SEs associated with drug resistance in small cell lung cancer, revealed the drug resistance mechanisms of SEs, and provided insights into the clinical applications of SEs.

**Electronic supplementary material:**

The online version of this article (10.1186/s12920-019-0520-9) contains supplementary material, which is available to authorized users.

## Background

Small cell lung cancer (SCLC) is an aggressive type of malignant tumor that shows rapid recurrence after chemotherapy and accounts for approximately 15% of all lung cancers. SCLC is the most destructive subtype of lung cancer and often exhibits neuroendocrine features [1]. Although SCLC is initially sensitive to chemotherapeutic drugs, rapid development of drug-resistant disease and a lack of existing therapies after relapse have led to poor outcomes in patients with this disease. Despite intensive efforts by clinical and basic investigators, the vast majority of drugs for SCLC are unsuccessful in the clinical setting. Therefore, to obtain more effective drugs, there is an urgent need to elucidate the drivers of therapeutic resistance in recurrent SCLC.

Genomic alterations, particularly gene amplifications, confer SCLC with the ability to acquire chemoresistance quickly. However, the underlying mechanisms of specific resistances remain unknown. The extent to which rapid acquisition of drug resistance after initial treatment is dependent on gene amplification in SCLC is not yet known. Gene amplification is usually modulated by the *trans*-regulatory apparatus and cis-regulatory elements [2], such as transcription factors (TFs) and super enhancers (SEs), which significantly increase the frequency of gene transcription. Gene transcription is the first step in gene expression; during this process, RNA polymerase, together with one or more TFs, binds to a particular segment of DNA. TFs are proteins that bind to DNA and regulate gene expression by suppressing or promoting transcription. The particular region of DNA to which TFs bind is called the promoter, enhancer, or SE. SEs, a key class of noncoding DNA *cis*-regulatory elements, have been the focus of recent studies in the field of cancer research and were first reported by Richard et al. [3]. SEs are screened through a three-step process: First, enhancer loci are represented as peaks on chromatin immunoprecipitation (ChIP)-seq data. Second, enhancers within 12.5 kb of each other are merged into stitched enhancer regions. Third, the ChIP-seq signal for each enhancer region (both stitched and single enhancers) is calculated. All enhancer regions are ranked along the X axis on the basis of the signal enrichment plotted on the Y axis. The signal value obtained at the inflection point of the resulting curve with a slope of 1 is the threshold between the SE and the general enhancer. SEs are designated as regions to the right of the inflection point of the curve [4].

Although the total number of regulatory elements may be in the millions, only a few hundred SEs control key genes, giving each cell its own unique identity and functions. Accordingly, SEs have been shown to be involved in oncogene activation in cancer [5]. Moreover, SEs play major roles in pathological changes, various diseases, cell type-specific development and differentiation, and chemoresistance acquisition [6, 7]. Therefore, SEs can serve as useful biomarkers for tracking and understanding the evolution of cancers and ultimately may be important targets for intervention therapy [8]. Mutiple co-activators are often required for SEs to function appropriately [9]. However, it is unclear how and what factors can efficiently promote enhancer activation and drug resistance. Therefore, a better understanding of genetic alterations and expression regulation is crucial for identifying new therapeutic targets.

Here, we aimed to establish a universal approach for identifying functional units of SEs linked to chemoresistance and their target genes. We also predicted transcriptional factors targeting these SEs in chemoresistant H69AR SCLC cells. Our findings provide perspectives for the use of SEs as targets to develop a novel disease therapeutic schedule and establish new directions for the clinical analysis of drug resistance.

## Methods

### Chromatin immunoprecipitation-sequencing (ChIP-seq)

2 × 10^7^ cells were fresh harvested and fixed in 1% formaldehyde/medium buffer for 10 min at room temperature. Fixation was stopped by addition of glycine to a final concentration of 125 mM. Fixed cells were washed three times with PBS buffer, and centrifuged (5000 rpm, 5 min). Pelleted cells and pulverized tissues were lysed in 100 ml 1% SDS lysis buffer and sonicated to 150–300 bp using a Bioruptor (Diagenode). ChIP was performed using the following antibodies: H3K27ac (ab4729, Abcam). After recovery of ChIP and input DNA, whole-genome-amplification was performed using the VAHTS Universal DNA Library Prep Kit (Vazyme) and VAHTS Universal Adapter (Vazyme). Amplified DNAs were purified by PCR purification columns (TIANGEN). Thirty nanograms of amplified DNA was used for each sequencing library preparation (Vazyme) and sequenced on NovaSeq (Illumina) to an average depth of 40 million reads per library.

### Sequence mapping and ChIP-seq density analysis

Sequence reads were mapped against human reference genome (hg19) using bowtie2 (version 2.2.9), allowing only one mismatch in the seed. The parameters were set as ‘–n 1–k1’, ‘samtools (version 1.3.1) view –bS’ and ‘bamflag (version 2.1) –u –m 3’. Only the uniquely mapped reads were kept to peak calling by MACS (version 1.40) with default parameter ‘-g mm –B –S --call-subpeaks’ [10]. Gene Interval Notator implement in CARPET was used to annotate peaks over RefSeq human genes [11]. A peak was assigned to the transcriptional start site (TSS) of a RefSeq gene when falling into the surrounding 4 kb (±2 kb). Promoters were defined as 6 kb regions (±3 kb) surrounding the TSS. To reduce background signals, read densities of each ChIP library were revised against the input library. Peaks with significant ChIP enrichment relative to the input library were detected using CCAT (version 3) (FDR < 5%). Peak densities within a region were computed by counting the total number of mapped reads normalized by the library and region size, a metric equivalent to reads per million mapped reads per kilobases (RPKM).

### Identification of predicted super enhancers

Predicted enhancers were determined as enriched H3K27ac regions at least 2 kb from annotated TSS. Distal predicted enhancers displaying high H3K27ac signals were recommended as mistaken predictions and thus left out analyses. Predicted enhancers were further subdivided into typical enhancers or predicted super-enhancers using the ROSE algorithm. The regions of predicted super-enhancer with at least one base overlap across multiple GC lines were incorporated using BEDTools, and predicted enhancers localizing to regions difference from the predicted super-enhancer regions were classified predicted typical enhancers. The presence of predicted typical or predicted super-enhancers in individual samples was decided by the level of H3K27ac enrichment according to background (*P* < 0.01, empirical test). To assign predicted super-enhancers/enhancers to genes, we calculated distances from the predicted super-enhances/enhancer centre to the nearest activated TSS, defined as a promoter (500 bp flanking aside TSS) with H3K27ac enrichment above random chosen regions.

### RNA-seq and analysis

Total RNA was extracted from H69 and H69AR by RNAiso Plus (Takara Bio, Dalian, China). 1 μg of RNAs each group used to the library construction by using the VAHTS mRNA-seq v2 Library Prep Kit for Illumina® (Vazyme, NR601) following the manufacturer’s instruction as detailed in the Additional file 1 section.

### Functional enrichment analysis

Gene functions and pathways, including super enhancer associated genes and differential expression genes, were analyzed several online databases. Gene Ontology (GO) analysis was performed for the target genes and DEGs with DAVID. *p*-value < 0.05 was set as the cutoff value. Kyoto Encyclopedia of Genes and Genomes (KEGG) Pathway (http://www.genome.jp/kegg) was also performed for these genes with KOBASS2.0. *p*-value < 0.05 was set as the threshold.

### TF binding motif analysis

We interrogated enrichments of TFs in predicted super-enhancers using the JASPAR database. Transcription factor binding sites with at least 60% of overlap with predicted super-enhancers were counted. Binding densities of TFs were computed as the total binding sites detected in the regions divided by the total size of the regions in unit of million base pairs (Mbp). The top TF identified from the outputs were used for expression correlation analysis. Additionally, we also identified binding motifs using MEME with JASPAR 2016.

### Cell, RNAi and transfections

Chemoresistance is the most important factor resulting in the death of cancer patients. Transcriptional regulation by super enhancer (SE) has an important role in the relapse of small cell lung cancer for acquired drug resistance. H69AR is the only resistant cell line for small cell lung cancer in the ATCC cell lines. So we selected the chemoresistant cell line H69AR and its parental cell line H69. Cells were transfected with validated siRNAs for SP1, IRF1, and FOXP1. Detailed description can be found in the Additional file 1 setion.

### RNA extraction and qRT-PCR

RNA isolation and Real time qRT-PCR were performed as manufacturer’s protocols, and as detailed in the Additional file 1 setion.

### In vitro proliferation assay and statistics

Cell counting kit 8 (CCK8) method were performed for in vitro proliferation assay using CCK8 kit (Dojindo) according to the manufacturer’s instructions. The GraphPad Prism 6 was used for statistical analysis. Data were calculated as mean ± SD. Student’s t-test was took to decide statistical significance.

## Results

### Screening of SEs in H69AR cells by chromatin immunoprecipitation-sequencing (ChIP-seq)

**Fig. 1:**
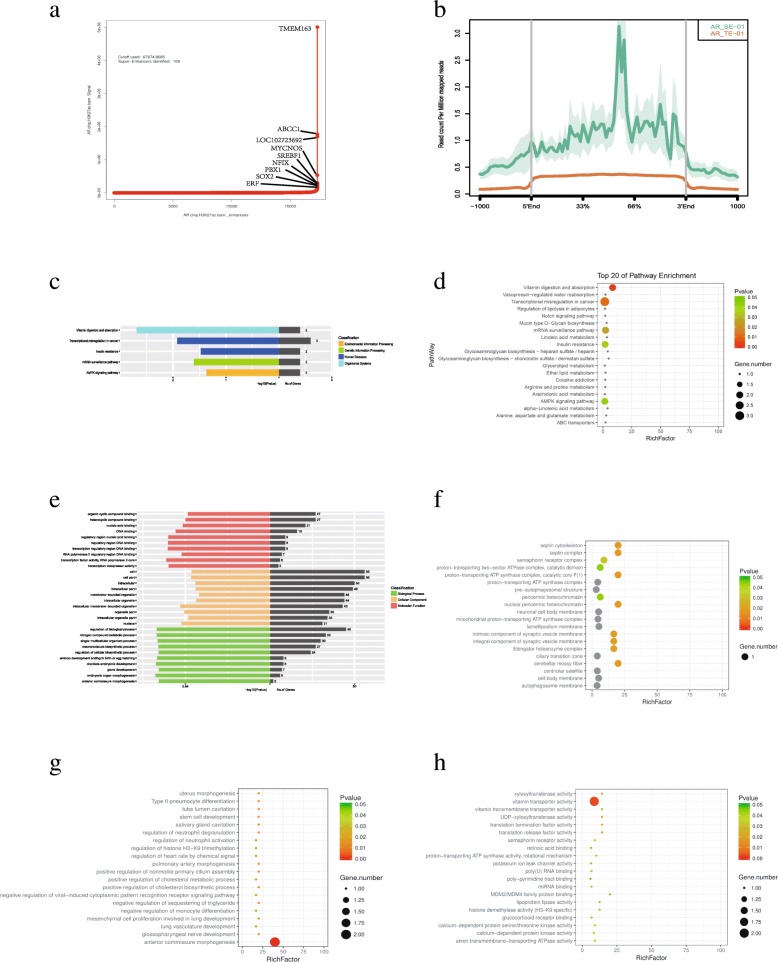
SEs in H69AR cells and bioinformatics analysis. **a**. Distribution of ChIP-seq density across enhancers with 108 SEs with a cutoff value of 67,674.8085. **b**. Read count per million mapped genes across SEs and TEs. AR: H69AR cells; SE: super enhancer; TE: typical enhancer. **c**. KEGG analysis of SE target genes. **d**. Top 20 enriched pathways in KEGG analysis for SE target genes. **e**. GO classification of SE target genes. **f**. Top 20 enriched pathways for cellular components. **g**. Top 20 enriched pathways for biological processes. **h**. Top 20 enriched pathways for molecular function

In total, 108 SEs were identified from H69AR cells using ChIP-seq with a cutoff value of 67,674.8 (Fig. 1a). Detailed information is shown in Table 1. These SEs spanned DNA domains whose median length was 89,584 bp, which was an order of magnitude higher than the typical enhancer (TE; median length: 1926 bp), and showed abundances that were at least one order of magnitude larger than that of the TE (Fig. 1b). Kyoto Encyclopedia of Genes and Genomes (KEGG) analysis showed that chemoresistance formation may be greatly influenced by pathways including vitamin and lipid metabolism and Notch and AMP-activated protein kinase (AMPK) signaling pathways (Fig. 1c, d). In addition, several genes involved in the insulin resistance pathway may have contributed to drug resistance (Fig. 1c, d).

**Table 1:** The detail information of super enhancers (Part of)

ID	Chrom	Start	End	Size	Associated gene	Method	Genome
SE_AR_002	chr16	16,036,480	16,138,317	101,837	ABCC1	H3K27ac	Human (hg19)
SE_AR_003	chr16	17,251,168	17,509,936	258,768	LOC102723692	H3K27ac	Human (hg19)
SE_AR_004	chr2	16,079,293	16,091,717	12,424	MYCNOS	H3K27ac	Human (hg19)
SE_AR_006	chr17	17,584,709	17,879,534	294,825	SREBF1	H3K27ac	Human (hg19)
SE_AR_007	chr17	55,925,998	56,035,303	109,305	CUEDC1	H3K27ac	Human (hg19)
SE_AR_008	chr16	85,228,407	85,522,422	294,015	MIR5093	H3K27ac	Human (hg19)
SE_AR_009	chr16	17,147,700	17,180,155	32,455	LOC102723692	H3K27ac	Human (hg19)
SE_AR_010	chr19	13,043,361	13,209,040	165,679	NFIX	H3K27ac	Human (hg19)
SE_AR_016	chr2	133,023,162	133,030,668	7506	ANKRD30BL	H3K27ac	Human (hg19)
SE_AR_017	chr17	79,844,783	80,024,031	179,248	ASPSCR1	H3K27ac	Human (hg19)
SE_AR_018	chr18	76,363,629	76,593,274	229,645	SALL3	H3K27ac	Human (hg19)
SE_AR_031	chr1	164,527,778	164,621,592	93,814	PBX1	H3K27ac	Human (hg19)
SE_AR_032	chr3	181,404,844	181,478,727	73,883	SOX2	H3K27ac	Human (hg19)
SE_AR_083	chr19	42,720,575	42,808,008	87,433	ERF	H3K27ac	Human (hg19)
SE_AR_088	chr1	16,465,999	16,509,151	43,152	EPHA2	H3K27ac	Human (hg19)
SE_AR_094	chr5	92,898,999	92,957,936	58,937	NR2F1	H3K27ac	Human (hg19)
SE_AR_095	chr1	23,875,382	23,895,952	20,570	ID3	H3K27ac	Human (hg19)
SE_AR_107	chr19	2,013,379	2,065,005	51,626	MKNK2	H3K27ac	Human (hg19)

Next, we performed gene ontology (GO) analysis of the associated genes (Fig. 1e–1h). From the results of bioinformatics analysis, we found that the majority of genes belonged to the cellular components and/or molecular functions cluster (Fig. 1e). These results demonstrated that chemoresistance formation was accompanied by changes in cellular components and biological processes, such as metabolism remodeling [12]. In pathways enriched in biological processes, we chose to focus on cholesterol metabolism-related processes in addition to some lung development processes (Fig. 1f) because the results suggested that cholesterol metabolism may contribute to chemoresistance formation. Moreover, histone H3K9 trimethylation was also identified by bioinformatics analysis (Fig. 1f); this mechanism may play a vital role in inhibiting the expression of tumor-suppressor genes and pro-apoptotic genes to facilitate drug resistance. In pathways enriched in cellular components, we found that synaptic vesicles may also play significant roles in drug resistance, including the transport of the ATP synthase complex, which has a key role in ATP-binding cassette (ABC) C1-related multidrug resistance [13] (Fig. 1g). In pathways enriched in molecular functions, we found that vitamin transporter activity was significantly enriched in H69AR cells, as were the functions of calcium protein kinase and histone H3K9 (Fig. 1h).

In summary, we not only screened 108 SEs associated with chemoresistance but also proposed a possible mechanism through which acquisition of chemoresistance was driven by vitamin and lipid metabolism remodeling induced by an anticancer drug. Moreover, our results suggested that the statuses of H3K9 demethylase and trimethylation could have significant effects on chemoresistance in SCLC.

### Analysis of chemoresistance associated genes using RNA-seq

H69AR cells were derived from H69 cells cultured in the presence of adriamycin with increasing concentrations for 14 months. These cells are almost 50-fold resistant to adriamycin compared with the parental cell line H69. In order to elucidate the gene expression profiles of H69AR and H69 cells, we used RNA-seq technology to obtain differentially expressed genes (DEGs) related to drug resistance.

**Fig. 2:**
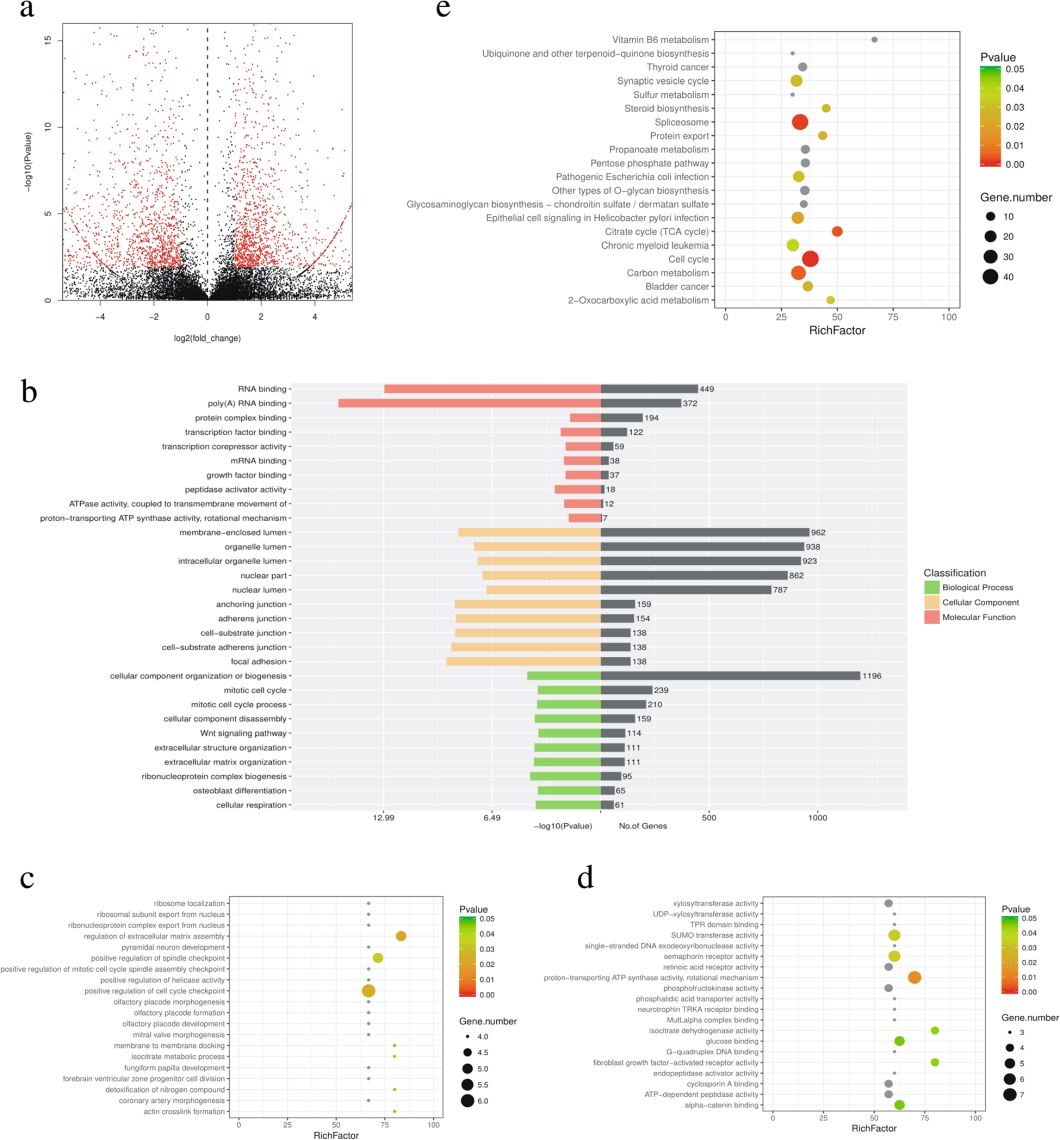
Bioinformatics analysis of differentially expressed genes between H69AR and H69 cells. **a**. Volcanic map of DEGs between H69AR and H69 cells. DEGs: differentially expressed genes. **b**. GO classification of DEGs. **c**. Top 20 enriched pathways for biological processes. **d**. Top 20 enriched pathways for molecular function. **e**. Top 20 enriched pathways by KEGG analysis

From the RNA-seq data, we characterized 1623 significantly upregulated DEGs in H69AR cells compared with H69 cells (Fig. 2a, Table 2). These DEGs were functionally annotated by GO and KEGG analyses (Fig. 2b–e). In the GO analysis, many genes were classified into the lumen and cell substrate junction, which may be related to the extracellular matrix remodeling in drug resistance acquisition (Fig. 2b). Additionally, many genes were assigned to cellular component organization or biogenesis and extracellular matrix organization in biological processes (Fig. 2b). These results demonstrated that during the acquisition of drug resistance in cancer cells, cellular component organization and biogenesis as well as extracellular organization were altered. In the molecular function analysis, most of the genes were involved in RNA binding and transcription-associated binding subtypes (Fig. 2b), which enabled cancer cells to respond quickly to anticancer drugs. In the subtype of molecular function, most genes were classified into RNA and protein complex binding (Fig. 2b). Thus, their functions were mainly related to transcriptional regulation.

**Table 2:** Differential expressiongenes between H69AR and H69 (Part of)

Gene	H69AR	H69	Log2(Fold_change)	p-value
HOXB9	228.338	0	−12.88396701	1.01E-24
COL1A2	344.62	0	−12.02077079	1.59E-42
HEY1	30.795	1.89376	−4.023370716	3.75E-08
ABCC1	1306.69	9.0491	−7.173926888	1.39E-297
NID1	85.2176	0	−10.77771999	4.00E-14
FBN2	15.9688	0	−10.45043797	0.000781566
MOXD1	36.0964	0	−11.39282462	2.94E-06
SOX2	243.046	0	−11.06258103	1.03E-35
MGC4473	84.163	0	−10.94412969	1.24E-13
FGFR1	40.8157	0	−7.867646656	2.40E-10
SLC12A7	17.4803	0	−10.15018672	0.000322703
MNX1	16.2196	0	−10.02136625	0.00046936
MYCN	895.032	0	−10.02116417	7.65E-149
LTBP1	26.4505	0	−10.00719865	7.78E-06
IFITM1	111.329	0	−9.934176564	2.91E-20
CDC42EP1	36.7847	0	−9.930180176	1.14E-07
SOX9	7.9101	0	−3.983695933	0.005427113
SP8	34.8206	0	−9.636102719	1.37E-07
SYT17	16.4721	0	−9.592478478	0.000278129
NEDD9	61.9691	0	−9.590789312	1.78E-12
DLX2	39.202	0	−9.527478125	1.77E-08
NEURL1B	15.9639	0	−9.294082989	0.000260711
NFIX	86.0713	0	−8.87827816	3.14E-18
NOTCH1	16.3472	0	−7.69294596	5.34E-05
EFHD1	144.371	0	−10.86427096	1.48E-22
VIM	837.548	4.25909	−7.619482847	4.68E-185
NR2F1	57.9309	0	−7.901400818	4.95E-14
HES1	64.7715	0	−7.272795449	2.86E-16
ABCC1	1306.69	9.0491	−7.173926888	1.39E-297
NFIC	69.2684	0	−7.140562658	1.93E-17
DDIT4	287.096	45.9881	−2.642200731	4.48E-45

From pathway enrichment in GO analysis, five pathways were identified, including extracellular matrix assembly, cell cycle checkpoint, proton-transporting ATP synthase, fibroblast growth factor-activated activity, and SUMO transferase (Fig. 2c, d). Interestingly, spliceosome, cell cycle, carbon metabolism, and citrate cycle pathways were significantly enriched in KEGG analysis (Fig. 2e). In addition, other important pathways were also identified as follows: synaptic vesicle cycle, steroid biosynthesis, protein export, and epithelial cell signaling (Fig. 2e).

According to bioinformatics analysis of RNA-seq data, most DEGs were roughly classified into transcriptional regulation, cellular components, extracellular matrix reorganization, glucose and lipid metabolism, and proton-transporting ATP synthase, which functions as a member of the superfamily of ABC transporters involved in multidrug resistance [14]. Our findings also suggested that spliceosome, lumen, and fibroblast growth factor-activated pathways may contribute to the onset of chemoresistance. Importantly, the RNA-seq data showed good correlations with genome-wide histone modifications.

### Identification of SEs related to chemoresistance

**Fig. 3:**
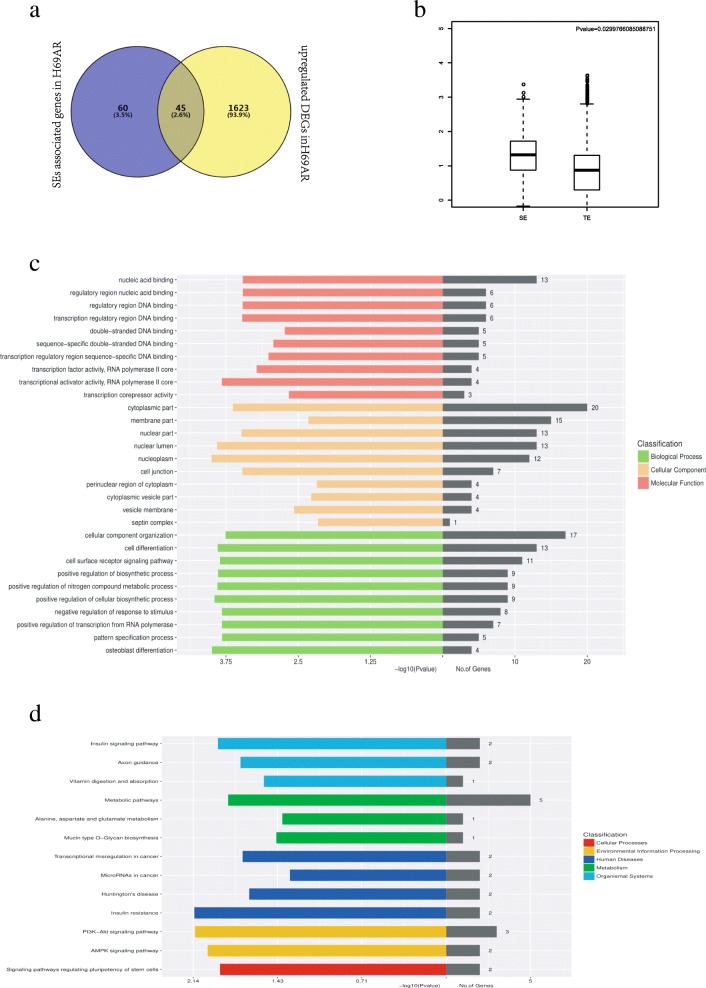
Screening and analyzing of chemoresistance-associated SEs. **a**. The intersection of SE-associated genes and upregulated DEGs. **b**. Box plot of the expression of genes from SEs and TEs (*P* < 0.05). **c**. GO classification of SE-associated upregulated DEGs. **d**. KEGG analysis of SE-associated upregulated DEGs

To investigate the roles of SEs in acquisition of chemoresistance, we examined the intersection of 108 specific SE-associated genes identified by ChIP-seq analysis for H3K27ac (Table 1), combined with 1668 upregulated genes from RNA-seq data in H69AR cells and parental H69 cells (Table 2). We obtained 45 genes by Venny (http://bioinfogp.cnb.csic.es/tools/venny/; Fig. 3a), almost 40% of which may be related to chemoresistance. Moreover, a comparison of tag densities at SEs and TEs demonstrated significant enrichment at the former (Fig. 3b). These genes were also classified by GO and KEGG analyses (Fig. 3c, d).

From the results of GO analysis, the functions of screened SE-associated genes were mainly related to DNA binding and transcriptional activator activity of RNA polymerase II (Fig. 3c). These findings implied that SEs increased the expression of target genes, which served as transcriptional factors regulating downstream gene expression, and suggested that SEs are related to various physiological and pathological conditions. In the classification of biological processes, there were three main processes for acquisition of chemoresistance: biosynthetic and metabolic processes, cell surface receptor signaling pathways, and RNA polymerase transcriptional regulation. Additionally, KEGG analysis showed that metabolism- and insulin-related pathways may play vital roles in modulating the acquisition of chemoresistance. The phosphatidylinositol 3-kinase/Akt and AMPK signaling pathways were also identified as being related to the regulation of drug resistance (Fig. 3d).

### Master TFs predicted by the SEs using MEME-ChIP

**Fig. 4:**
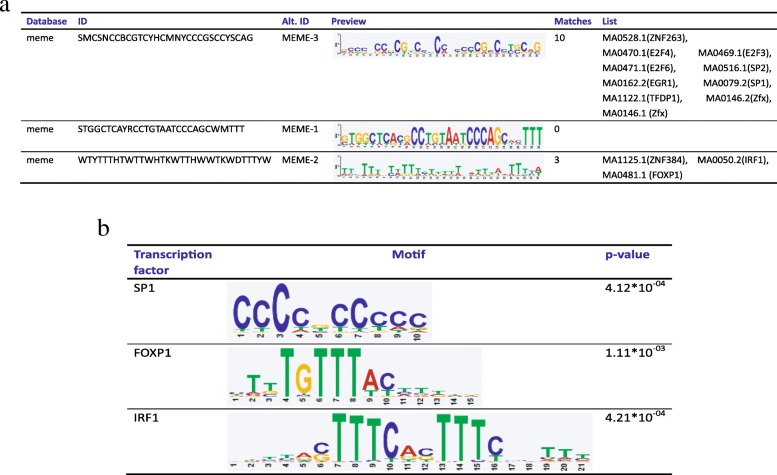
Master TF binding to SEs predicted by MEME. **a**. Table depicting TF binding motifs enriched at constituent enhancers within SE regions were predicted by MEME. **b**. Detailed information of selected transcription factor binding motifs and associated *p* values

SEs maintain cell identity, and SE-driven transcription alters not only protein-coding genes but also noncoding regulatory elements, thereby contributing to the cancer cell state [3, 15]. SE-driven transcription regulation involves highly organized interactions between the transcriptional machinery and TFs, particularly master TFs [16, 17]. Thus, we applied these SEs to predict their master TFs using MEME-ChIP [18]. Twelve master TFs were predicted (Fig. 4a). Subsequently, we performed functional validation of these predicted master TFs (Fig. 4b).

### Verification the relationship between master TFs and chemoresistance

To determine the function of predicted master TFs, we selected Foxp1, IRF1, and SP1 among the 12 enriched master TFs and analyzed the relationships between TFs and drug resistance.

#### Forkhead box P1 (FOXP1) and chemoresistance

**Fig. 5:**
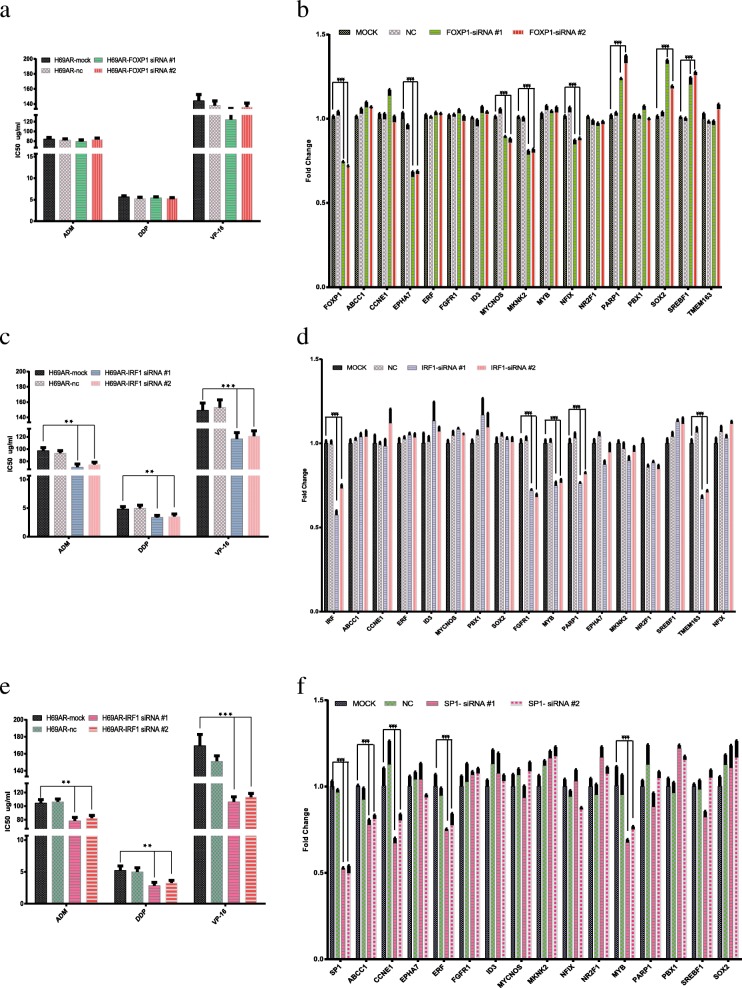
CCK8 assays and qRT-PCR analyses in cells transfected with siRNAs targeting FOXP1, IRF1, and SP1. **a**. Changes in IC_50_ after FOXP1 knockdown. **b**. Changes in gene expression following knockdown of FOXP1. **c**. Changes in IC_50_s after IRF1 knockdown. **d**. Changes in gene expression following knockdown of IRF1. **e**. Changes in IC_50_s after SP1 knockdown. **f**. Changes in gene expression following knockdown of SP1

The transcriptional repressor FOXP1 has been defined as a tumor suppressor in several cancers. To test the effects of decreased FOXP1 expression on chemoresistance, we reduced the level of FOXP1 expression in H69AR cells via RNA interference. Then, we analyzed FOXP1-knockdown cells to assay drug resistance to adriamycin, cisplatin, and etoposide byCell Counting Kit-8 (CCK8) assays. The results demonstrated that the half-maximal inhibitory concentration (IC_50_) of H69AR cells transfected with FOXP1 siRNA was not significantly altered compared with that in untransfected H69AR cells (*p* > 0.05; Fig. 5a).

In order to determine whether FOXP1 is related to drug resistance, we used quantitative real-time reverse transcription polymerase chain reaction (qRT-PCR) to detect the expression levels of related genes. Our results showed that SP1 knockdown in H69AR cells significantly reduced the expression levels of *EPHA7*, *MYCNOS*, *MKNK2*, and *NFIX* mRNAs (Fig. 5b). In contrast, the levels of *SREBF1*, *PARP1*, and *SOX2* mRNAs were upregulated (Fig. 5b). The protein products of the *PARP1* and *SOX2* genes have been shown to be involved in chemoresistance [19]. Thus, our results showed that FOXP1 had a self-contradictory role in chemoresistance. Additional studies are needed to elucidate the molecular mechanisms of the dual roles of FOXP1-regulated gene expression.

#### IRF1 and chemoresistance

IRF-1 is a crucial TF that regulates the immune response, immune cell development, cell growth, tumor suppression, autophagy, and apoptosis in mammalian cells. Therefore, we next evaluated the roles of IRF1 in chemoresistance. CCK8 assays showed that the IC_50_ values of H69AR cells transfected with IRF1 siRNA were significantly altered compared with those in untransfected H69AR cells (*p* < 0.01; Fig. 5c). Next, using qRT-PCR, we showed that some critical genes, i.e., *FGFR1*, *MYB*, *PARP1*, and *TMEM163* were upregulated (Fig. 5d). These results indicated that IRF1 may coordinate with MYB to target *FGFR1*, *PARP1*, and/or *TMEM163* to facilitate drug resistance. Further studies are needed to determine the mechanisms through which IRF1 promotes chemoresistance.

#### SP1 and chemoresistance

SP1 is an extensively studied TF that is important for cell growth, differentiation, apoptosis, and carcinogenesis. To determine the effects of SP1 knockdown on chemoresistance, we knocked down SP1 expression in H69AR cells via RNA interference. We then analyzed drug resistance in SP1-knockdown cells. The results demonstrated that knockdown of SP1 promoted chemotherapy sensitivity to an anticancer drug (Fig. 5e). Moreover, SP1 knockdown H69AR cells significantly reduced the expression levels of *ABCC1*, *CCNE1*, *ERF*, and *MYB* mRNAs (Fig. 5f). Overall, these results suggested that SP1 activated *ABCC1* and stimulated the expression of the *CCNE1*, *ERF*, and *MYB* genes, which have been shown to be related to chemoresistance and cancer relapse [20–22]. However, the underlying mechanisms through which SP1 targets *ABCC1* via *CCNE1*, *ERF*, and *MYB* are still unclear.

## Discussion

Chemoresistance is a major factor contributing to death in patients with cancer and is therefore an urgent problem in clinical treatment. Transcriptional regulation by SE is current research hotspots and has been shown to have an important role in the relapse of small cell lung cancer owing to acquired drug resistance. H69AR cells are currently the only chemoresistant small cell lung cancer cell lines available from ATCC. Therefore, in this study, we screened SEs associated with chemoresistance using the chemoresistant cell line H69AR.

We used ChIP-seq analysis to identify 108 SEs in the SCLC chemoresistant cell line H69AR. We found 45 SEs that may be related to drug resistance. SEs activate transcription and show greater sensitivity to perturbation than TEs according to size, content, and TF density. Indeed, inhibition of the transcriptional machinery is required for the assembly and maintenance of SEs, resulting in inhibition of tumor growth, oncogenic transcription, and cancer evolution, including acquisition of chemoresistance. Such dependence on SE-driven transcription for chemoresistance hinders the therapeutic management of drug resistance. Despite numerous clinical trials, the therapeutic regimen for patients with SCLC has not improved significantly in decades. The application of next-generation sequencing technologies has facilitated screening of many SEs associated with chemoresistance in SCLC. Thus, our findings provided an excellent opportunity to reveal the drug resistance mechanisms and provide new therapeutic strategies for the clinical application of SEs.

SEs are important regulatory elements affecting gene expression and are occupied by the mediator and master regulators, also known as master TFs [23]. A small number of master TFs, typically called lineage regulators, are adequate to establish regulation of gene expression patterns that define cell identity [16]. The master TFs in any one cell type can aggregate at the enhancers of many active cell-type master genes and may contribute to the organization of master gene expression patterns [24]. Master TFs bind coordinately to enhancer DNA elements and assemble co-activators to the transcriptional machinery [25, 26]. The close relationship between transcriptional deregulation and cancer evolution is highlighted by the observation that many oncogenes and tumor-suppressor genes encode TFs, strongly implying that altered gene regulation is a fundamental mechanism of cancer progression, including acquisition of chemoresistance [27]. In this study, we predicted 12 master TFs that were found to bind to the SEs and activate targeting genes. Subsequently, we selected three predicted TFs to examine the relationships of these TFs with chemoresistance. From these results, we found that some TFs may have dual roles in chemoresistance. For example, FOXP1 not only reduced the expression of chemoresistance-associated genes, such as *MKNK2* and *NFIX*, but also stimulated the expression of other genes, including *SOX2*, *SREBF1*, and *PARP1* [28, 29]. These results implied that drug resistance is a highly complicated process and that multiple targets may been to be combined to treat chemoresistance in patients with cancer.

High-throughput sequencing data from H69AR and H69 cells showed that most DEGs were classified into transcriptional regulation, cellular components, extracellular matrix reorganization, and glucose and proton-transporting ATP synthase, a member of the superfamily of ABC transporters. Additionally, our results highlighted the involvement of the spliceosome, lumen, fibroblast growth factor-activating, lipid metabolism, vitamin metabolism, steroid biosynthesis, and histone H3K9 trimethylation pathways. Accordingly, we expect that our findings may provide insights into the mechanisms of acquired drug resistance.

In conclusion, our results provided perspectives on the use of SEs as biomarkers to develop novel therapeutic tools for overcoming chemoresistance in patients with SCLC. However, there is insufficient genetic evidence demonstrating their unique molecular functions and the mechanisms underlying the regulation of gene expression; thus, many follow-up studies are required. First, it will be necessary to demonstrate experimentally whether the 12 master TFs actually binding to the SEs. According, we plan to use CRISPR/Cas9 technology to knock out these SEs and then detect changes in corresponding target genes by qRT-PCR. We will then use ChIP assays with antibodies to these master TFs. After ChIP experiments, the binding DNA sequence will be detected using high-throughput sequencing. Second, although our findings provide insights into drug resistance, further studies are needed to validate the presence of SEs in additional lung cancer cell lines. However, H69AR cells are the only chemoresistant small cell lung cancer cell line currently available from ATCC. Thus, we used this cell line to screen for SEs, and we believe that the results were reliable and scientifically sound. If we were to use drugs to induce chmoresistance in other cell lines, insufficient induction may lead to the selection of enhancers that are not closely related to actual drug resistance. Moreover, such drug-induced cell lines may increase noise during bioinformatics analysis of sequencing data and prevent detection of many drug-related enhancers. Accordingly in our future studies, we will use Cas9 technology to study the relationships among specific enhancers, target genes, major transcription factors, and drug resistance in cell lines other than H69AR. We expect that the use of CRISPR/Cas technology will facilitate the regulation of these SEs and their corresponding genes. From these studies, we expect to improve our understanding of the relationships between master TFs and their target SEs.

## Additional file

Additional file 1: Supplementary Materials and Methods. (DOCX 19 kb)

## Abbreviations

ADM: Adriamycin
ChIP-seq: Chromatin immunoprecipitation followed by deep sequencing
DDP: cisplatin
FOXP1: Forkhead box P1
IRF1: Interferon regulatory factor 1
SE: Super enhancer
siRNA: small interference RNA
SP1: Specificity protein 1
TE: Typical enhancer
TF: Transcription factor
VP-16: etoposide
