# Supplementary material for: Integrated high-throughput analysis identifies super enhancers associated with chemoresistance in SCLC
Source: BMC Med Genomics. 2019 May 22;12:67. doi: 10.1186/s12920-019-0520-9 (PMC6532255; doi:10.1186/s12920-019-0520-9)
Supplement: Supplementary file 1 — Supplementary Materials and Methods. (DOCX 19 kb) [file 12920_2019_520_MOESM1_ESM.docx]

**Supplementary Materials and Methods**

**RNA-seq and analysis**

Total RNA was extracted from H69 and H69AR by RNAiso Plus (Takara Bio, Dalian, China). 1μg of RNAs each group used to the library construction by using the VAHTS mRNA-seq v2 Library Prep Kit for Illumina ® (Vazyme, NR601) following the manufacturer’s instruction. Firstly, using poly-A oligo-attached magnetic beads, mRNA was purified from the total RNA, followed by RNA fragmentation using divalent cations in Vazyme Frag/Prime Buffer. The first strand cDNA synthesis using reverse transcriptase and random primers. Second strand cDNA synthesis was afterwards performed using buffer, dNTPs, DNA polymerase I and RNase H. After that, cDNA fragments were end repaired by the addition of a single 'A' base at the 3' -end of each strand, linked with the special sequencing adapters (Vazyme, N803). The products were purified and size selected using VAHTSTM DNA Clean Beads (Vazyme, N411) in order to obtain the suitable size (350–450bp) for sequencing. The prior concentrations of the cDNA libraries were decided using the Qubit ® RNA Assay Kit on the Qubit ® 3.0 Flurometer, while the insert size was determined using the Agilent Bioanalyzer 2100 system. Samples with the proper insert size were accurately quantified using qPCR on the Step One Plus Real-Time PCR system (ABI, USA). Then, clustering of the index-coded library samples was accomplished on a cBot Cluster Generation System (Illumina, USA) in line with the manufacturer's instructions, followed by sequencing on an Illumina Hiseq 2500 platform with 150bp paired-end module performed by Vazyme BioTechnologies CO. Ltd (Nanjing, JiangSu, China). Image analysis and base calling was obtained by the Illumina GA processing pipeline. Gene expression values were measured in FPKM units. Differential expression between groups was identified as genes showing altered expression by at least two-fold and absolute differences of 0.5 FPKM.

**Cell, RNAi and transfections**

Chemoresistance is the most important factor resulting in the death of cancer patients. Transcriptional regulation by super enhancer (SE) has an important role in the relapse of small cell lung cancer for acquired drug resistance. H69AR is the only resistant cell line for small cell lung cancer in the ATCC cell lines. So we selected the chemoresistant cell line H69AR and its parental cell line H69. Cells were transfected with validated siRNAs for SP1, IRF1, and FOXP1. SiRNA transfections were executed using Lipofectamine 3000 (Invitrogen, ) according to manufacturer's protocols. These siRNAs sequences would be seen in Table 1. All cells were cultured in RPMI1640 with 10% fetal bovine serum (FBS, Gibco ) at 37℃ in 5% CO2.

Table 1 The sequences of FOXP1, IRF1 and SP1 siRNAs

| siRNA | Sense sequence (5'-3') |
| --- | --- |
| FOXP1-siRNA #1 | GCAGCAACCACUUACUAGATT |
| FOXP1-siRNA #2 | GUACAGCCCAAUGUAGAGUTT |
| IRF1-siRNA #1 | CAGAUUAAUUCCAACCAAATT |
| IRF1-siRNA #2 | GUGAUCUGUACAACUUCCATT |
| SP1-siRNA #1 | CAGAUAUCGAGGAGGUGAATT |
| SP1-siRNA #2 | GCUCAAGGCAUGAUUCCAATT |

**RNA extraction and qRT-PCR.**

Total RNA was extracted from H69 and H69AR by RNAiso Plus (Takara Bio, Dalian, China). RNA was reverse transcribed by FastKing one step RT-PCR kit (KR123, TianGen, Beijing, China). Real-time PCR was performed by Talent fluorescence quantitative detection kit (SYBR Green) brought from Tiangen of China. All real time PCR values of each gene were normalized to beta-actin. The relative expressions of these genes were calculated by the 2^-△△Ct^ method. Primer sequences can be seen in Table 2.

Table 2 Primersequences of associated genes

| Gene | Direction | Sequence (5'-3') |
| --- | --- | --- |
| ABCC1 | Forward primer | GCCGATGGCTCCGACC |
|  | Reverse primer | ACGAGGACCGTGTTCTGAAA |
| ID3 | Forward primer | TGCCCACTTGACTTCACCAA |
|  | Reverse primer | AGGCCACAAGTTCACAGTCC |
| SOX2 | Forward primer | ATGGACAGTTACGCGCACAT |
|  | Reverse primer | CGAGCTGGTCATGGAGTTGT |
| NFIX | Forward primer | GCCGGGTGCTTCAGATCAAT |
|  | Reverse primer | TGCTTGATCTCGGGCTTCTC |
| ERF | Forward primer | GTGCGATATTAACCCGGGAGG |
|  | Reverse primer | TCTGGCTTGTAGGCCCAATC |
| NR2F1 | Forward primer | CCCCCTTCGTATTCTTCTTATCGT |
|  | Reverse primer | CTCTTCAGCCGGCAACCAAC |
| PBX1 | Forward primer | CCAAGCTAACTCGCCCTCAA |
|  | Reverse primer | CCTGCCAACCTCCATTAGCA |
| MYCNOS | Forward primer | AGACAACCGCACTCGCAG |
|  | Reverse primer | CTGTCTGTGTTTGAGCTGTCG |
| MKNK2 | Forward primer | CTGCAGAGGTGGGACAGTCA |
|  | Reverse primer | ACATTAACACATGGCCGTTCAC |
| SREBF1 | Forward primer | CTGCTGACCGACATCGAAGA |
|  | Reverse primer | TGGGTCAAATAGGCCAGGGA |
| CCNE1 | Forward primer | GCAGGATCCAGATGAAGAAATG |
|  | Reverse primer | TAATCCGAGGCTTGCACGTT |
| EPHA7 | Forward primer | CAGCTACAGCTGTCTCCAGT |
|  | Reverse primer | CACAGTGCCTTCTCCCAATGA |
| PARP1 | Forward primer | AGCGTGTTTCTAGGTCGTGG |
|  | Reverse primer | CATCAAACATGGGCGACTGC |
| IRF1 | Forward primer | AAAGTCGAAGTCCAGCCGAG |
|  | Reverse primer | TGTTGTAGCTGGAGTCAGGG |
| FOXP2 | Forward primer | CCGGCGAACGGCAAA |
|  | Reverse primer | CTTCCTCTTACAAACTTTCGGGT |
| TMEM163 | Forward primer | GTAAGACCCCTCTCCCCATTTC |
|  | Reverse primer | CCGGGTACTCCAAAATGTCA |
